# Climatological influences on major storm events during the last millennium along the Atlantic coast of France

**DOI:** 10.1038/s41598-020-69069-w

**Published:** 2020-07-21

**Authors:** Pierre Pouzet, Mohamed Maanan

**Affiliations:** 1 UMS CNRS 3281 OSUNA-OR2C-UMR CNRS 6554, LETG, Université de Nantes, Bâtiment IGARUN, 1 rue de la Noë, 44300 Nantes, France; 2 UMR CNRS 6554 LETG-OR2C-UMS CNRS 3281 OSUNA, Université de Nantes, Bâtiment IGARUN, 1 rue de la Noë, 44300 Nantes, France

**Keywords:** Climate and Earth system modelling, Palaeoclimate, Environmental impact, Natural hazards

## Abstract

This paper reviews the climatological influences on major past storm events in the North-east Atlantic. Analyses are based on a millenary record of sedimentological and historical impacts affecting coastal societies. The effects of 20 past storms have been found from sedimentary deposits from the last 1,000 years. Historical archives confirmed these events. This paper highlights five major storms that have markedly impacted coastal populations. They date back to 1351–1352, 1469, 1645, 1711 and 1751 AD. The 1351–1352 AD event is defined as a millennium storm that was “likely apocalyptical”, provoking serious damage and long lasting floods on much of the European coast. Major storm impacts have mostly been recorded during positive North Atlantic Oscillation phases. Four decreasing temperature phases are concomitant with 1300–1355, 1420–1470, 1560–1590 and 1690–1715 AD periods, during which much of the northern Atlantic coast of France underwent severe storm damages.

## Introduction

Reconstructing extreme storm history is a methodological challenge of importance in order to understand climate change^1,2^. Data studying recent storms have proved that anthropogenic warming has impacted extratropical storm activity or trajectories in the North Atlantic Ocean^3,4^. These studies are mainly based on isotopic temperature evolution or oceanological and climatological modelling. Historical and sedimentological data are used to understand centuries-old historical storm trends, thus extending this analysis’s timescale. They give us valuable information on past storm dynamics, including their relation with various climatological mechanisms, such as the North Atlantic Oscillation (NAO) or temperature variation^5–7^. The most impacting storms studied can testify to notable ancient phases of high extreme storm activity. These storm phases have deeply impacted their environments, coastal societies and the area’s past economic activity. This paper reviews storm impacts recorded on central European coasts; many of which have been largely studied by historians, thanks to an extensive and high-quality regional archive collection^8–14^.

Sedimentological data are primarily used in storm research to expose environmental disturbances of Storm Events (StE) that occurred during the last millennium^7,15^. Several sedimentological palaeostorm studies have already been conducted on the central European coast^16–20^. However, they mainly focus on Holocene storm phases or post nineteenth century storms. While a large collection of historical documents precisely exposing storms’ societal and economic impacts is available, sedimentological records of millennium storms are still less documented in this area. Consequently, this paper has three main objectives: (1) to detect impacting StE in the last 1,000 years thanks to a combination of sedimentological data and historical documents; (2) to use this crossing to spatialise damage and assess past trajectories of the most disturbing storms that crossed the area; and (3) to compare storm chronologies with NAO and temperature variations to consider their possible climatological influences.

## Results

### Study area

Back barrier lagoons, which are separated from the sea by a sandy barrier, are relevant environments to detect past StE^15,21^. The Petite Mer de Gâvres (PMG) and the Traicts du Croisic (TDC) lagoons were selected along the French Atlantic coast as they are environments with a constant natural evolution and with no human impacts^22^. Since the area is threatened by coastal flooding, they are localised in a fitting place to detect past extratropical storms^9^. This coast has a semi-diurnal tidal regime and the highest tidal ranges near these two sites reach ∼ 6–7 m. Only past storms combined with high tides can be observed.

**Figure 1:**
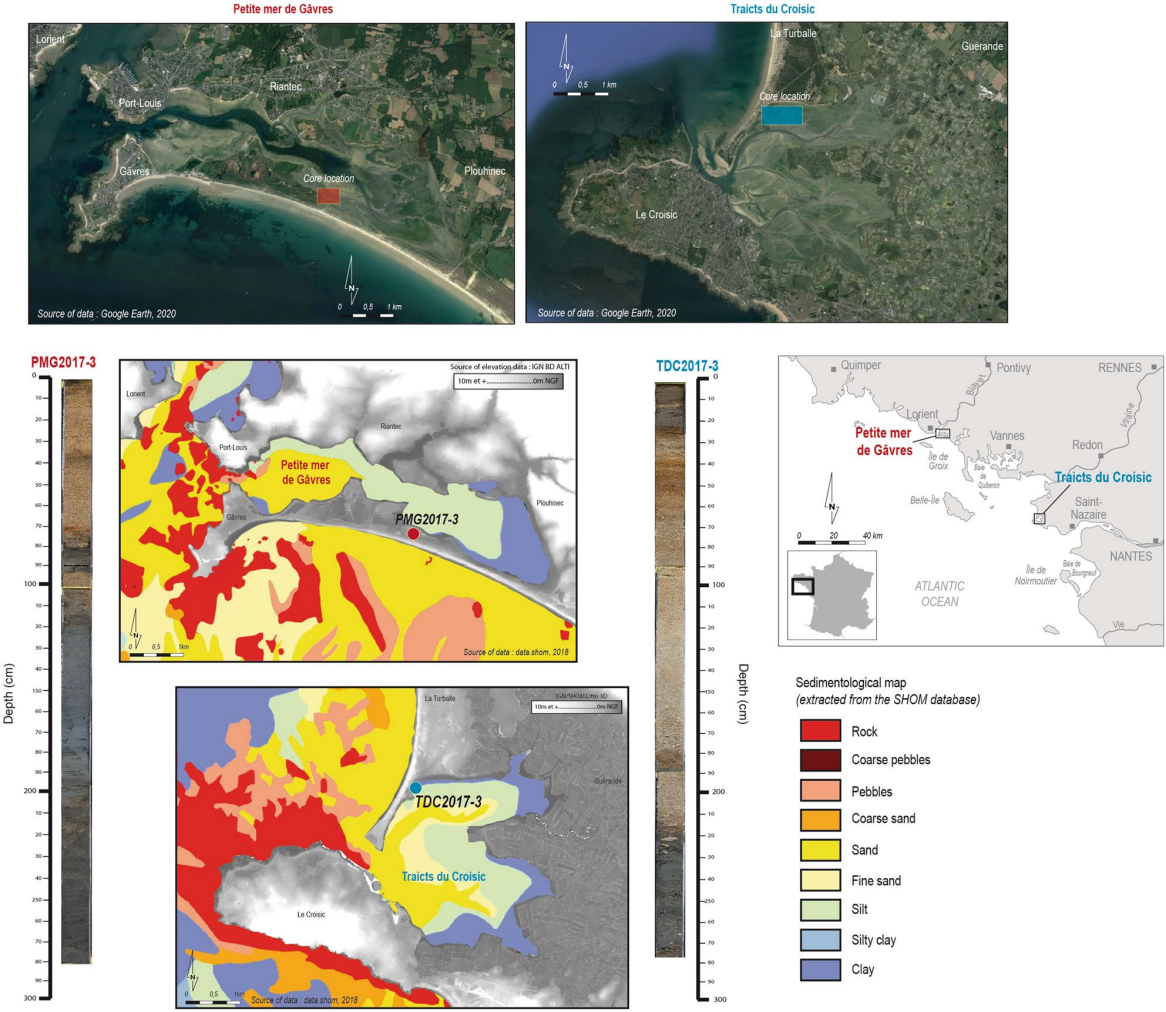
Characteristics of the two studied sites. The two aerial photographs were obtained from Google Earth. Map was generated with QGIS 2.18.28 from the QGIS Development Team (2020), QGIS Geographic Information System, Open Source Geospatial Foundation Project (https://qgis.osgeo.org).

The Petite Mer de Gâvres (PMG) lagoon is localised near the Blavet Estuary, known as the Rade de Lorient, in southern Brittany (Fig. 1). A 6-km long dune barrier, formed during the Holocene glacial retreat, separates the lagoon from the sea and connects the Gâvres tombolo to the continent^23^. Most of the PMG undergoes the tidal range, coming from a 300 m wide pass situated at the north-west. The PMG is formed of two basins created by the progression of two narrow sandy peaks in the centre of the lagoon. The clayey bottom of the lagoon has been selected, as it testifies to calmer sedimentological transport. We cored a 3 m deep core (PMG2017_3), located about 400 m from the channel to avoid both urbanisation and potential erosion due to tidal retreat. At 100 km south, the Traicts du Croisic (TDC) is in the Pays-de-la-Loire region (Fig. 1). This lagoon in closing is connected to the sea by a 500 m wide pass separating the Pen Bron spit and the Croisic peninsula, which is mainly formed of gneiss and granites^24^. Two wide main channels supply the two basins and bring marine sediments from the inlet localised at the south-west. The northern basin is separated from the sea by the 1 km long Pen Bron spit. The closest areas to the channel are composed of sandy deposits. Farther from the channels, these deposits turn into silt, then clay, as marine dynamics subside. A 3 m deep core (TDC2017-3) has been collected in a clayey environment, at 5 m from the dune, behind its thinner part.

### Sedimentological evidence of past storm impacts

**Figure 2:**
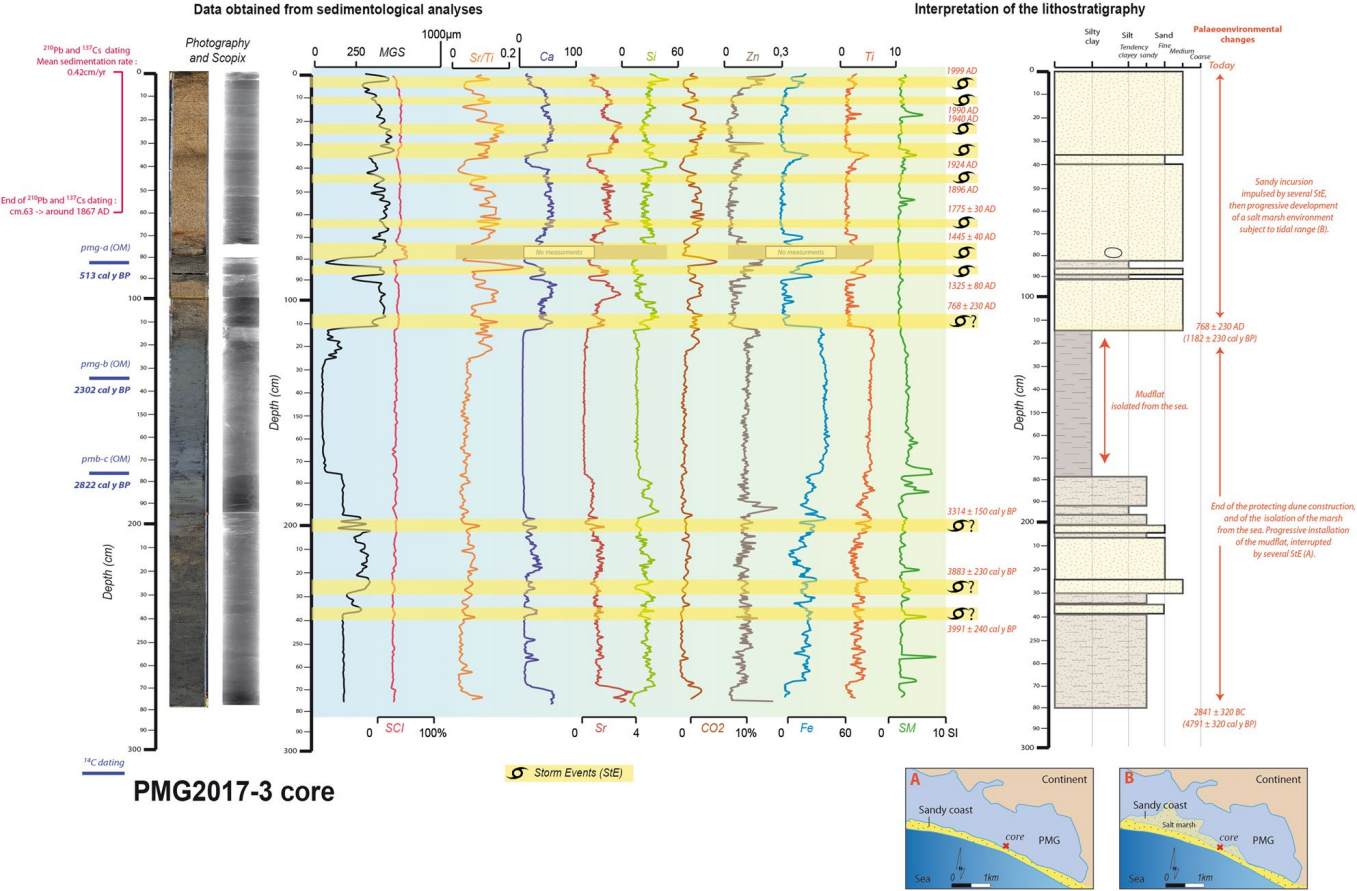
Log of the Petite Mer de Gâvres core (PMG2017-3), with palaeoenvironmental reconstruction. The maps were generated with Adobe Illustrator CS5 (https://www.adobe.com/).

**Figure 3:**
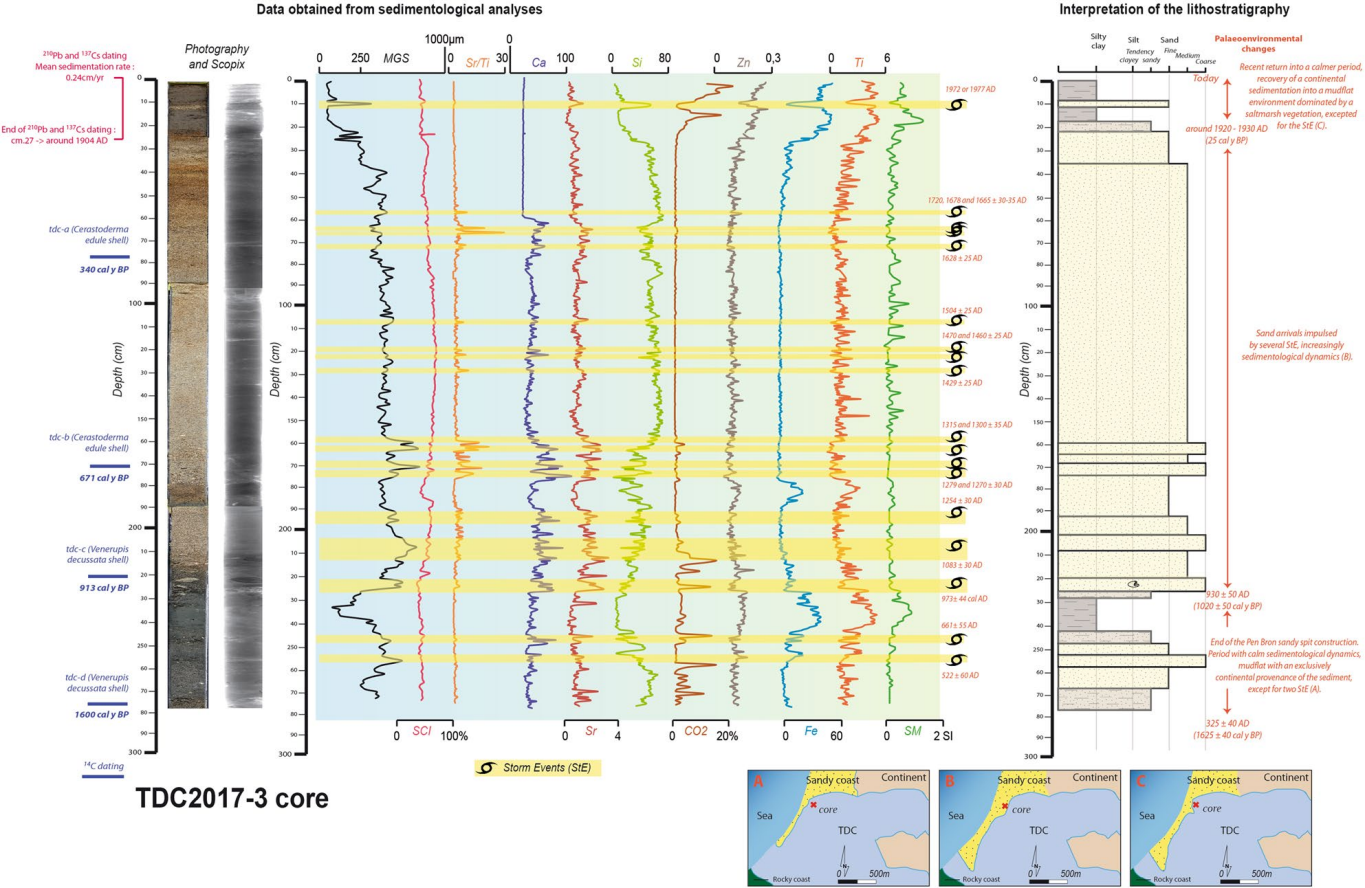
Log of the Traicts du Croisic core (TDC2017-3), with palaeoenvironmental reconstruction. The maps were generated with Adobe Illustrator CS5 (https://www.adobe.com/).

Storm conditions can cause overwash processes over the protecting dune of both back barrier depositional environments^5,25^. Marine allochthonous layers detected after coring sampling can be considered as “washover” deposit. They testify to sedimentological parameters which are distinct from the coastal lagoonal sequences^15^. The marine or coastal origin of each layer identified is obtained from radiography, grain size, geochemical, colour, organic matter and Magnetic Susceptibility (MS) analyses. Sediments are then dated by radiocarbon (^14^C), ^210^Pb and ^217^Cs isotopes to assess the deposition date of each extracted washover deposit. High values of marine proxies [Mean Grain Size (MGS), colour (and especially lightness from SCI), strontium (Sr), calcium (Ca) and silicon (Si)] combined with low values of coastal or continental indicators such as Organic Matter (OM: CO_2_ values), Zn (zinc), Fe (iron), Ti (titanium) and MS indicate a marine origin of the sediment. In the PMG and the TDC, 8 and 15 marine incursions are extracted; respectively (Figs. 2, 3). The complete lithostratigraphy analyses of both coastal lagoons are detailed in the Supplementary S1 section. The crossing with historical archives testifies to the storm origin of each washover deposit extracted in the sedimentological cores^17^.

**Figure 4:**
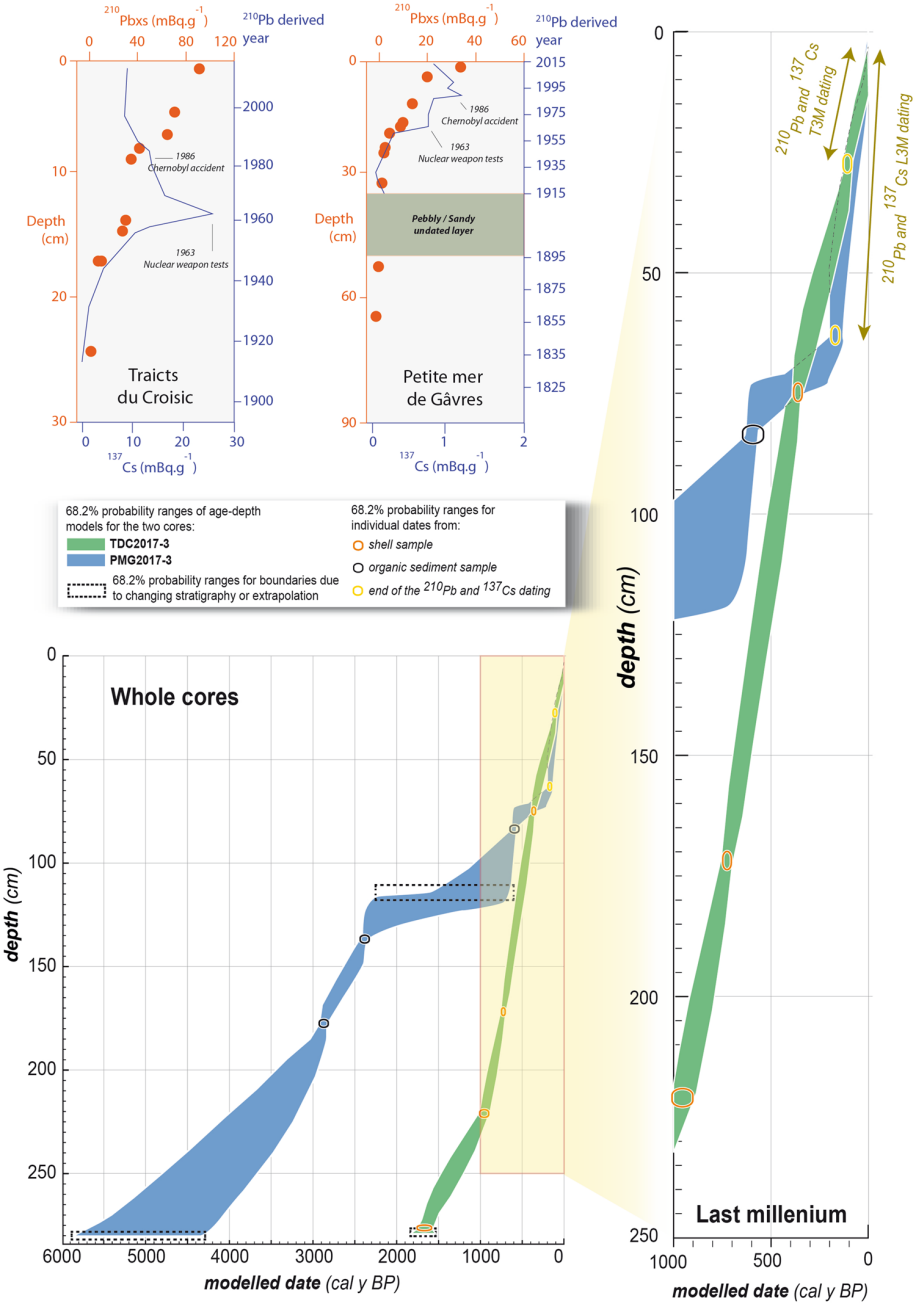
Radiocarbon age-depth models obtained using the OxCal P_Sequence algorithm for the two investigated sites, with ^210^Pb and ^137^Cs dating curves.

Both dating sequences reach good overall agreement indices. Each radiocarbon sample has been dated with a low uncertainty (Table 1), resulting in two accurate age-depth models (Fig. 4). The TDC2017-3 and the PMG2017_3 cores show low uncertainties in the upper 230 cm and 100 cm; respectively, which correspond to the last millennium. The transition between ^210^Pb/^137^Cs and ^14^C dating at 27 cm depth is well defined in TDC2017-3. In the PMG2017_3 core, however, a higher margin of error can be found at the 800–1000 cal y BP (950–1150 AD, for *Anno Domini*) period. It corresponds to the limit of two different layers. The 70–80 cm depth section also shows higher uncertainties (reaching ± 80 years) due to the ^210^Pb/^137^Cs until ^14^C dating methods transition. Nevertheless, these two curves provide precise information to date recorded environmental changes during the last millennium. From the combination of these dating methods, a mean sedimentation rate of 0.22 cm/year (TDC) and 0.12 cm/year (PMG) is estimated since 1000 AD. These results are consistent with other sedimentation rates found in the western French back-barrier environments, which are mainly assessed between 0.1 and 0.5 cm/year^26–28^.

**Table 1:** Sample details, results of AMS radiocarbon dating and calibration for the Petite Mer de Gâvres (PMG2017_3) and the Traicts du Croisic (TDC2017-3) cores.

No.	Lab. no.	Sample name	Sample type	Age ^14^C (BP) or pMC *Dates calibrated with Marine13 curve and local marine reservoir effect ∆R = 151 ± 45^67^	Ranges of calendar age for 68.2% and 95.4% confidence levels
1	GdA-5375	PMG2017_30.2/83 cm	Organic matter	465 ± 25	68.2% probability 1428 AD (68.2%) 1446 AD 95.4% probability 1414 AD (95.4%) 1454 AD
2	GdA-5378	PMG2017_30.3/135 cm	Organic matter	2,295 ± 25	68.2% probability 398 BC (68.2%) 370 BC 95.4% probability 405 BC (84.2%) 356 BC 284 BC (9.4%) 254 BC 246 BC (1.8%) 236 BC
3	GdA-5376	PMG2017_30.4/178 cm	Organic matter	2,730 ± 30	68.2% probability 901 BC (68.2%) 836 BC 95.4% probability 930 BC (95.4%) 812 BC
4	GdA-5371	TDC2017-3.01/78 cm	*Cerastoderma edule* shell	905 ± 30*	68.2% probability 1500 AD (68.2%) 1620 AD 95.4% probability 1465 AD (95.4%) 1660 AD
5	GdA-5372	TDC2017-3.3/172 cm	*Cerastoderma edule* shell	1,240 ± 20*	68.2% probability 1250 AD (68.2%) 1340 AD 95.4% probability 1215 AD (95.4%) 1404 AD
6	GdA-5373	TDC2017-3.4/221 cm	*Venerupis decussata* shell	1,460 ± 40*	68.2% probability 1036 AD (68.2%) 1166 AD 95.4% probability 980 AD (95.4%) 1239 AD
7	GdA-5374	TDC2017-3.6/276 cm	*Venerupis decussata* shell	2,235 ± 30*	68.2% probability 224 AD (68.2%) 370 AD 95.4% probability 148 AD (95.4%) 420 AD

**Figure 5:**
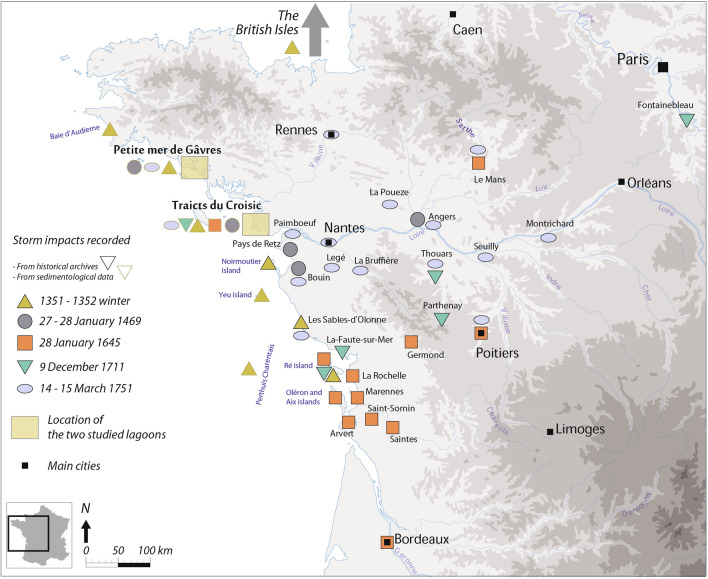
Maps of impacts recorded in sedimentological and historical archives for the five main studied storms. This map was generated with QGIS 2.18.28 from the QGIS Development Team (2020), QGIS Geographic Information System, Open Source Geospatial Foundation Project (https://qgis.osgeo.org); and Adobe Illustrator CS5 (https://www.adobe.com/).

Twenty past storm events have been extracted in the last 1,000 years. Fifteen storms present local impacts. The recent 1896, 1924, 1940, 1972 (or 1977), 1990 and 1999 AD storms have impacted north-west France^17^. Older dates are estimated at 1628 ± 24, 1504 ± 25, 1460 ± 25, 1429 ± 25, 1300 ± 35, 1279 ± 30, 1270 ± 30, 1254 ± 30 AD and 1083 ± 30 AD. They may correspond to the storms which hit the European Atlantic coasts on 1 February 1632, 23 November 1509, in October 1457 and on 4 March 1408^29^. The four thirteenth century events are part of a common phase of stormy activity dated around 1250–1350 AD, which also includes the 1351 AD storm. The 1083 AD marine incursion can be linked to a stormy period which impacted north-west France around 1050–1085 AD^30^. Including the 1351 AD storm, the five remaining events have deeply impacted the European Atlantic coast. Precise information on their damage, reported in Table 2 and mapped in Fig. 5, is presented in historical documents and detailed in the following sections. Dates are expressed in the Gregorian calendar and the new style is used for pre-October 1582 AD historical dates.

**Table 2:** Storm impacts recorded in sedimentological data for the five main storm events highlighted, dated at 1351–1532, 1469, 1645, 1711 and 1751 AD.

Storm mentioned	Location	Impact	Data used	Source
1351–1352 AD	Petite Mer de Gâvres	Marine deposit dated 1325 ± 80 AD	Sedimentology	This study
1351–1352 AD	Traicts du Croisic	Marine deposit dated 1315 ± 35 AD	Sedimentology	This study
1351–1352 AD	Yeu Island	Marine deposit dated 600–500 cal y BP (1350–1450 AD)	Sedimentology	Pouzet et al.^6^
1351–1352 AD	Baie d’Audierne	Marine deposit dated 1335 AD	Sedimentology	Van Vliet Lanoe et al.^20^
1351–1352 AD	Pertuis Charentais	Coarse grained sedimentation pulse	Sedimentology	Poirier et al.^19^
1351–1352 AD	NW Europe	European Atlantic Stormy Event estimated 600–300 cal y BP (1350–1650 AD)	Sedimentology/bibliography	Pouzet et al.^6^
1351–1352 AD	NW Europe	Storminess Event estimated 600–300 cal y BP (1350–1650 AD)	Sedimentology/bibliography	Sorrel et al.2012^7^
1351–1352 AD	British Isles	Storm impacts phase between 700 and 550 cal y BP (1250–1400 AD)	Several geological analyses/bibliography	Devoy et al.^31^, Hansom and Hall^32^, Oldfield et al.^33^ and Wilson et al.^34^
1351–1352 AD	Outer Hebrides, (Scotland)	High period of sand mobilisation between 692 and 504 cal y BP (258–1446 AD)	Sedimentology	Gilbertson et al.^35^
1469 AD	Petite Mer de Gâvres	Marine deposit dated 1445 ± 40 AD	Sedimentology	This study
1469 AD	Traicts du Croisic	Marine deposit dated 1470 ± 25 AD	Sedimentology	This study
1469 AD	NW Europe	European Atlantic Stormy Event estimated 600–300 cal y BP (1350–1650 AD)	Sedimentology/bibliography	Pouzet et al.^6^
1469 AD	NW Europe	Storminess Event estimated 600–300 cal y BP (1350–1650 AD)	Sedimentology/bibliography	Sorrel et al.^7^
1645 AD	Traicts du Croisic	Marine deposit dated 1665 ± 30 AD	Sedimentology	This study
1645 AD	NW Europe	European Atlantic Stormy Event estimated 600–300 cal y BP (1350–1650 AD)	Sedimentology/bibliography	Pouzet et al.^6^
1645 AD	NW Europe	Storminess Event estimated 600–300 cal y BP (1350–1650 AD)	Sedimentology/bibliography	Sorrel et al.^7^
1711 AD	Traicts du Croisic	Marine deposit dated 1720 ± 30 AD	Sedimentology	This study
1751 AD	Petite Mer de Gâvres	Marine deposit dated 1775 ± 30 AD	Sedimentology	This study
1751 AD	Traicts du Croisic	Marine deposit dated 1720 ± 30 AD	Sedimentology	This study

### Reconstructing environmental and socio-economic impacts of five major historical storms

Two notable impacts can be found in both cores around 1350 AD. They can be detected because of the 1325 ± 80 AD PMG high increases of MGS and marine elements (186 cm) and the 1315 ± 35 AD TDC important marine intrusion (162 cm). Other sedimentological evidence has been found in the European Atlantic coast: a Yeu Stormy period (1350–1450 AD), during a European Atlantic Stormy Event^6^ (1350–1650 AD EASE 1), a storm found at the Baie d’Audierne near 1335 AD^20^ and a “coarse grained sedimentation pulse” (CSP) detected near 1300 AD in the Pertuis Charentais^19^. At a larger scale, this impact concurs with a 1350–1650 AD European high storminess period^7^. In the British isles, damage was recorded between 1250–1400 AD^31–34^, with a peak of sand mobilisation between 1258–1446 AD^35^. Historical documents concur since they provide records of a damaging storm during winter 1351–1352 AD (Fig. 5). Numerous lowlands of Noirmoutier Island were flooded for nearly half a century^9^. Marshes were also destroyed in Olonne, and 15 years later, salt production was still impacted. This event has been defined as “likely apocalyptical” and as a major submersion of Ré Island^13^. Athimon presented this storm as “one of the most violent and dramatic meteorological events of the last millennium along the French Atlantic coast”^29^. Sedimentological and historical evidence highlight impacts from Brittany to the Pertuis Charentais. They also imply that this storm may have hit the British Isles, which would mean that much of the European Atlantic coast was affected.

Two other sandy inputs are reported in the PMG (1445 ± 40 AD at 79 cm depth) and the TDC lagoonal sequence (1470 ± 25 AD at 118 cm depth). A 3 cm diameter pebble of vein quartz has been extracted from this marine layer in the PMG core. This StE is part of two European Stormy Periods^6,7^. Impacts found in the TDC and PMG are linked to the storm that hit the French Atlantic coast during 27–28 January 1469 AD (Fig. 5). The tide coefficient is estimated around 106 (spring tide) during 28 January^29^. This 1469 AD storm seriously damaged dikes and salt marshes in Bouin. This former island was submerged overnight. Afterwards, historical records reported that 1500 tonnes of salt had probably been lost, thus inducing major economic losses^9^. Thirty metre long breaches appeared in the salt marsh causeways. Numerous ridges and roads were destroyed, and a salt merchant drowned. In the Retz region, marine flooding is recorded thanks to the taxes merchants had to pay to their lord (seigniorial taxes). Prices fell because of the impact of this storm^13,36^. The lands became sterile because of brutal marine incursions^8^. Windy impacts were recorded near Angers, where the bell tower of Saint-Aubin toppled, and “numerous trees” were uprooted^9^. The impacts were, for the most part, recorded on the coast (from mid-Vendee to southern Brittany). This is why the supposed trajectory of this event is more uncertain than in other cases. The damage reported around Angers attests to its possible eastern direction.

At 66 cm depth in the TDC core, a strong geochemical signal testifies to a marine incursion. It has been dated at 1665 ± 30 AD, which refers to the storm that hit much of France during 28 January 1645 AD (Fig. 5). It can be part of the two European Stormy Periods^6,7^. Marine flooding was reported in the marshes of Ré, Aix and Oléron islands, and in the cities of La Rochelle, Marennes, Arvert and Saint-Sornin^29^. A large ship collapsed into the lowlands situated at 3 m NGF (Nivellement général de la France) elevation, and nearly 30 ships sank. The losses in the salt marshes came to nearly “500,000 écus” (a French currency in the Middle-Ages) of salt lost. Inland, the Cathedral of Saintes was destroyed and the Notre Dame la Grande church of Poitiers was damaged. In Bordeaux and Le Mans, where the Sarthe River induced flooding because the heavy rain, two others churches were damaged. In Germond, many houses were destroyed. Presented in Athimon^29^, these impacts are extracted from the departmental archives of Charentes, Charentes-Maritimes, Gironde and Sarthe and historical local books^37–40^. This storm is qualified as a “disorder”, “horrible”, or “terrible” and much of the population hit by this event characterised it as “exceptional”^29^. Concerning the economic cost, it took three months for the Ré Island inhabitants to repair the damage, which impacted agricultural activities^41^. Overall, the spatial extent of this storm is considerable and includes much of the French Atlantic coast. An important concentration of storm impacts is identified between La Rochelle, Poitiers and Bordeaux. Bearing in mind the reports of wind destruction and river flooding in Le Mans, this event presents a SW–NE trajectory.

An important geochemical marine input, dated at 1678 ± 35 AD, is recorded at 64 cm depth in the TDC sequence. This environmental disturbance is linked to a storm that hit the central French Atlantic coast on 9 December 1711 AD (Fig. 5). A powerful storm surge was observed at La-Faute-sur-Mer^10^. Historical archives reported important damage, such as the destruction of seawalls and several saltmarsh flooding. The total cost is estimated at 1.19 million Euros at current currency values, mainly because repairing the dikes took a considerable amount of time. Ré island was seriously flooded^8^. After the storm, salt production tools were discovered near the forecourt of the church of The Portes en Ré^42^. According to local parish registers, this storm and its linked marine flooding made a deep impression on coastal families, which lasted for several centuries^8^. A written source called this event a “hurricane” after Thouars’s bell tower collapsed on the church’s vault: “a stone from the belfry was detached from the top, which fell on the vault of the choir (…) A few minutes after the top of the bell tower crossed the vault of the choir. Half of the bell tower collapsed falling on the arches of the choir and chapel of St. Margaret of Scotland” ^43^. The accident resulted in one casualty, named as “a beggar”. Similar bell tower damage is also reported in Parthenay, and this storm produced the most important “windthrow” damage of the eighteenth century in Fontainebleau^43^. This extratropical depression seems to have crossed central France in a SW–NE trajectory, without impacting Brittany. This hypothesis is attested by damage recorded in Fontainebleau forest^44^.

A last important historical storm was recorded in the TDC (1720 ± 35 AD at 57 cm depth) and the PMG (1775 ± 30 AD at 63 cm depth) cores. These two marine inputs are linked to the storm on 14–15 March 1751 AD (Fig. 5). At La Bruffière, a local parish register, written between 1751–1760^29^, presents the damage as: “A hurricane ripped out more than half of the church, (…), destroyed the nave of the church, (…) destroyed a large part of the houses, and ripped out and uprooted some stronger and taller oak trees (…). Finally, it is almost incredible the damage caused by this storm in this parish and others around.” Marine flooding was reported on the former island of Bouin and the Sables d’Olonnes. Wind impacts were recorded in Nantes and Thouars and according to the Francheteau family registers, Legé was severely impacted^9^. An ancient written source explains that “(…) about one hour after midnight, a storm began, or hurricane, that continued for a few hours with such violence that we did not know where to go, nor where to get to safety”^45^. Seuilly, Rennes, Le Mans, Poitiers and Angers churches or abbeys were partially destroyed^29^. Near Tours, the church of Saint-Croix of Montrichard was damaged (La Nouvelle République, August 2016). At La Ploueze, damage was so impressive that people thought this event happened at the same time as an earthquake^46^. Finally, this storm also had important impacts offshore. On 17 March 1751 AD, at the request of the Consulates, the Admiralty of Nantes issued an order directing all boats owners to unload their cargo within 24 h in order to leave as quickly as possible to Paimboeuf^8^. Their mission was to retrieve the goods and assist ships stranded by the storm. According to this source, the Admiralty wanted to reduce the damage of this event on trade^47^. The Parish register of Nantes reported that only three of the 60 ships remained unscathed, and many people were injured or died^29^. The numerous impacts recorded generally underline the eastern direction of this event.

## Discussion

Two main storm paths can be extracted from the spatial survey of environmental, societal and economic impacts recorded in the various archives and mapped on Fig. 5. The first one, regarding the 1469 and 1751 AD events, is estimated from west to east in a restricted part of the western Atlantic coast (from Vendee to southern Brittany). The second one seems to be more extended, with a SW-NE trajectory impacting a larger part of this coast. This second hypothesis is based on the analyses of the 1645 and 1711 AD events. These results concur with the study of Lozano et al.^48^. Four main stormy trajectories are exposed at the European scale, and two main storm paths are presented on the French Atlantic coast. Study cases of 1469 and 1751 AD seem to correspond to the trajectory “Zone 3” presented in the Fig. 4 of Lozano et al.^48^. The 1645 and 1711 AD storms are linked to the main extratropical cyclone track of “Zone 4”. Zone 3 events followed an eastward path nearly 45° N, and most of these storms occur west of 0°. The average lifespan of storm events is ∼ 4.5 days^49^ in the north-east Atlantic Ocean and ∼ 4.1 days in Zone 3^48^. Zone 4 extratropical storms follow a north-eastward direction from 15° W, for a mean ∼ 4.6 days lifespan^48^. Zone 3 and 4 storms present higher pressure values than Zones 1 and 2, which occur in northern Europe^48^. Extratropical cyclones trajectories vary with climate change, and a northward position has been observed due to recent global warming^3,4,50^.

**Figure 6:**
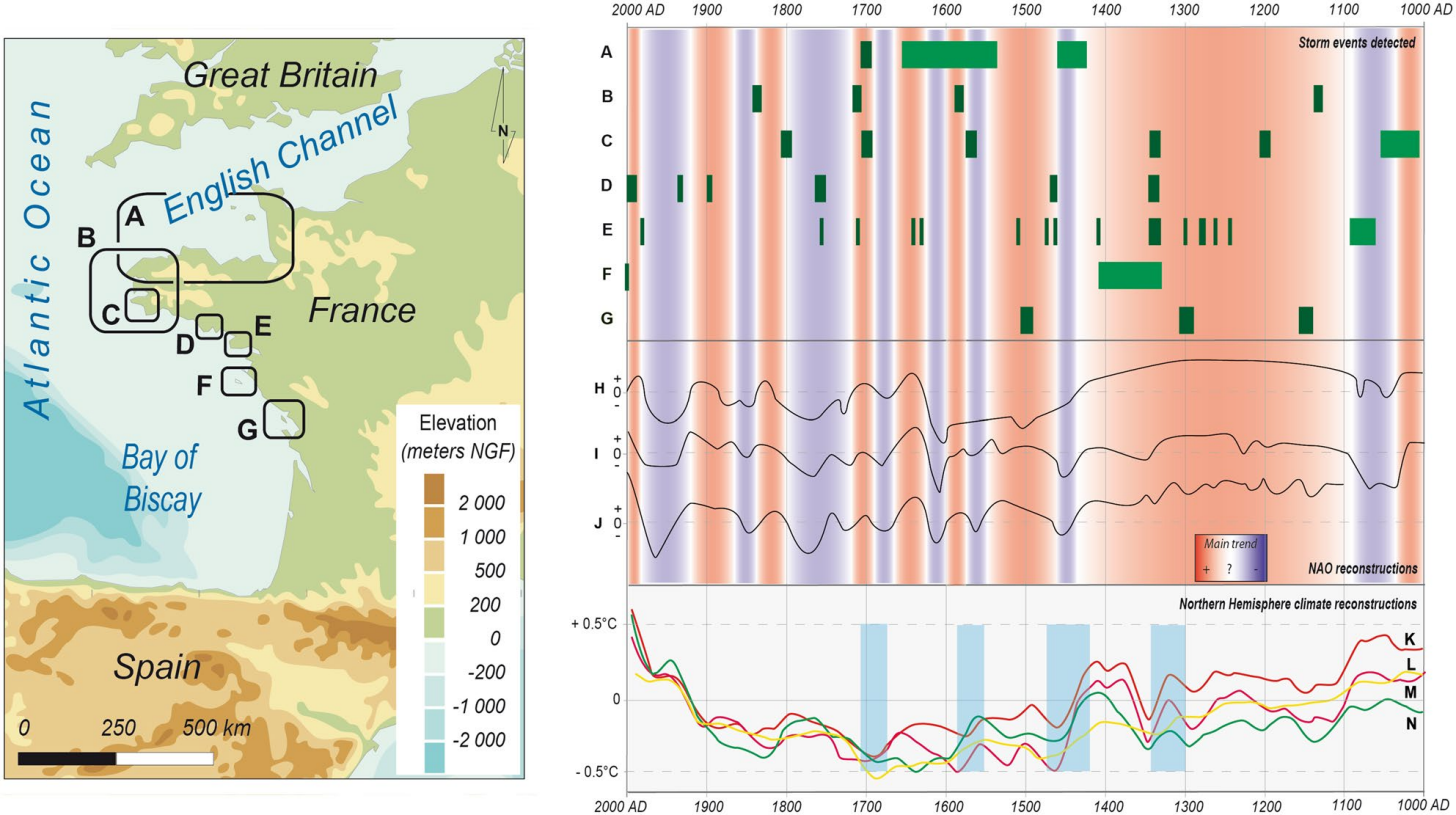
Synthesis of the sedimentological storm impacts review over the last millennium on the north of the French Atlantic coast, with climatological influences. Storm impacts from sedimentological studies along the NW French coast (Precise storm event dating in brown and estimated storm periods in green): A: North of Brittany and west Cotentin (Van Vliet Lanoe et al.^51^); B: NE and SW Brittany (Regnauld^52^); C: Audierne Bay (Van Vliet Lanoe et al.^20^); D: Petite mer de Gâvres (This study); E: Traicts du Croisic (This study); F: Yeu Island (Pouzet et al.^6^); G: Pertuis charentais (Poirier et al.^19^). Comparisons of the chronologies with three North Atlantic Oscillation reconstructions during the last millennium (H: Baker et al.^53^; I: Proctor et al.^54^; J: Trouet et al.^55^) with interpretation of the successive NAO positive phases in red and negative phases in violet. Comparisons of the chronologies with four temperature anomaly curves (from 1881 to 1980) extracted from the IPCC Fifth Assessment Report (Pachauri et al.^50^). Raw data are extracted from Pollack and Smerdon^59^ (K, red curve); Ljungqvist^57^ (L, yellow curve); Mann et al.^58^ (M, pink curve) and Hegerl et al.^56^ (N, green curve). Four phases of decreasing temperatures are displayed in blue, as they are linked to storm impacts recorded in several sites presented in the sedimentological review. The map was generated with Adobe Illustrator CS5 (https://www.adobe.com/).

Seven sedimentological studies presenting historical storm records in north-western France are summarised in Fig. 6. We recorded storm impacts in northern Brittany and west Cotentin^51^ (Fig. 6A). In Brittany, two previous studies recorded sedimentological storminess^52,20^, reported in Fig. 6B,C. Sedimentological storm impacts detected in the Petite Mer de Gâvres and the Traicts du Croisic are presented in Fig. 6D,E. The “Yeu Stormy Phase” A and a recent storm impact (Fig. 6F) are extracted from Yeu Island^6^. Finally, three “Coarse Grained Sedimentation Pulses” of the Pertuis Charentais, which are induced by storm activity^19^, are presented in Fig. 6G. These chronologies have been compared with the NAO variations over the last millennium. Three NAO reconstructions^53–55^ have been synthesised into an overall tendency (Fig. 6H,I,J). Phases of positive NAO state are assessed at 1000–1030, 1100–1430, 1450–1560, 1585–1600, 1640–1660, 1690–1710, 1870–1910 AD and 1990 AD to present. Storm impacts detected along the north of the French Atlantic coast presented in Fig. 6A–G are mostly recorded during NAO positive phases. Stormy phases have also been compared with four temperature curves of the northern hemisphere^56–59^, which were presented in the Intergovernmental Panel on Climate Change (IPCC) Fifth Assessment^50^ (Fig. 6K–N). Four periods present storm impacts in several sites reviewed in Fig. 6: 1300–1355 AD (Baie d’Audierne, TDC, PMG, Yeu Island and the Pertuis Charentais), 1420–1470 AD (Brittany-Cotentin, PMG and TDC), 1560–590 AD (the three northern references) and 1690–1715 AD (Brittany and TDC). From the IPCC Fifth Assessment temperature curves used, we noticed that these four storm phases are all concomitant with decreasing temperature periods. Storm impact may be influenced by a positive NAO state and a decreasing temperature phase in north-western France.

## Methods

### Sampling and sediment analysis

Both cores were collected using an Eijkelkamp 50 mm Ø, motor-driven percussion corer in the two coastal depositional environments (Fig. 1). A Trimble Differential Global Positioning System (DGPS) was used to survey core positions. Locations were linked to Institut national de l'information géographique et forestière (IGN) benchmarks and levelled according to the NGF datum. Cores were covered in long plastic tubes and stored at + 4 °C. Sediments were characterised by visual litho-microstratigraphic analysis to identify the main sedimentological changes, and to extract each detected macrofossil. Cores were cut into 1 cm long slices and oven-dried at 35–40 °C. A LECO carbon analyser estimated CO_2_ percentages after burning at 1,400 °C^60^. Grain size was measured using a laser granulometer (Malvern Mastersizer 2000). Geochemical elemental analyses were evaluated using an Avaatech© XRF core scanner at the UMR CNRS 5805 Environnements et Paléoenvironnements Océaniques et Continentaux. (EPOC) Laboratory (University of Bordeaux). Element intensities were normalised out of the total intensity^61^. Three marine elements (calcium, strontium, and less prominent silicon), and three terrestrial proxies (iron, titanium and less prominent zinc) were retained to interpret geochemical data^17,62^. The Scopix© system was used to take X-ray radiographs^63^. Each cm of both cores was meticulously examined. Lightness was estimated thanks to colorimetric analyses with a Minolta© Cm-2600d spectrometer^64^, and MS was measured using a Bartington MS2E-1©^65^.

### Dating

We used ^210^Pb and ^137^Cs dating for the upper part of the cores and ^14^C AMS dating for the lower part. Seven samples were dated in the Gliwice Absolute Dating Methods Center of the Institute the Silesian University of Technology (GADAM), by the accelerator mass spectrometry (AMS) technique. Organic sediment samples of high organic matter content were selected based on the CO_2_ percentage, and two well-formed marine shells were found in the TDC core. Organic samples were subjected to standard preparation to extract total organic carbon, which included treatment with 0.5 M hydrochloric acid, washing in demineralised water and drying. After CO_2_ combustion, graphitization took place in an automated graphitization system AGE-3^66^. Determinations of ^14^C content were carried out in the Direct AMS Laboratory, Bothell, USA. Radiocarbon ages were calibrated using the IntCal13 atmospheric curve^67^ for core PMG2017_3. As the TDC2017-3 is mainly composed of shell samples, the Marine13^67^ curve was used, with the local reservoir effect of 151 ± 45 years. The mean of the five nearest available data set from the Marine Correction Database points was calculated^68^. Results were introduced into the P_Sequence algorithm^69^ thanks to the OxCal software v.4.3.2^70^ in order to obtain the age-depth models. The collection year 2017 AD has been included at 0 cm depth, and the models were extrapolated to reach 280 cm depth for both cores (Fig. 4). The upper parts were precisely dated thanks to the ^210^Pb and ^137^Cs dating calculation in Pouzet et al.^17^. The two 1 m deep cores dated by ^210^Pb and ^137^Cs are localised a few metres from the two 3 m deep cores analysed in this study. According to Pouzet et al.^17^, a calculated 0.42 cm/year sedimentation rate was included in the upper 63 cm of the PMG age depth model. The 0.24 cm/year sedimentation rate calculated in the upper 27 cm of the TDC has also been included in the age-depth model.

## Supplementary information

Supplementary Information.

## Data Availability

The datasets generated during and/or analysed during the current study are available from the corresponding author on reasonable request.
