# Supplementary material for: Climatological influences on major storm events during the last millennium along the Atlantic coast of France
Source: Sci Rep. 2020 Jul 21;10:12059. doi: 10.1038/s41598-020-69069-w (PMC7374694; doi:10.1038/s41598-020-69069-w)
Supplement: Supplementary file 1 — Supplementary Information. [file 41598_2020_69069_MOESM1_ESM.pdf]

Climatological influences on major storm events during the last millennium along the Atlantic coast of France

Pierre Pouzet[1*] and Mohamed Maanan[2]

[1] UMS CNRS 3281 OSUNA – OR2C – UMR CNRS 6554 LETG
Université de Nantes, Bâtiment IGARUN, 1 rue de la Noë, 44300 Nantes, France.
+33 (0)2.53.48.76.75, pierre.pouzet@univ-nantes.fr
ORCID ID: 0000-0002-1988-8340

[2] UMR CNRS 6554 LETG – OR2C – UMS CNRS 3281 OSUNA
Université de Nantes, Bâtiment IGARUN, 1 rue de la Noë, 44300 Nantes, France.
+33 (0)2.53.48.76.57, mohamed.maanan@univ-nantes.fr
ORCID ID: 0000-0003-3526-2070

* Corresponding author. Phone: +33 (0)2.53.48.76.75. E-mail: pierre.pouzet@univ-nantes.fr

Supplementary information

S1. Detailed lithostratigraphy analysis of the two lagoons

The Petite Mer de Gâvres palaeoenvironment can be divided in two different stages (Figure 2). The first one is at the base of the core, between 115-280 cm (pre-768 ±230 AD; section A). It testifies to a calmer environment than the upper core. A silty environment between 180-280 cm is interrupted by several sandy StE. It characterises the end of the protecting dune construction, with notable grain size and geochemical variations. The mean sedimentation rate is estimated at 0.05 cm/yr. Three potential StE are extracted: 3314±150 cal y BP (1364 BC) at 205 cm, 3883±230 cal y BP (1933 BC) at 230 cm and 3991±240 cal y BP (2041 BC) at 238 cm. They expose peaks of Mean Grain Size (MGS), strontium (Sr), calcium (Ca), and decreases in iron (Fe) and titanium (Ti) concentrations. Dating uncertainty of these three potential StE is too high to confirm these hypotheses. Once the littoral spit formed, the dune transits until becoming a mudflat isolated from the sea, between 115-180 cm. This environment is composed of dense clays, rich in continental elements, with a mean sedimentation rate of 0.04 cm/yr. The second main stage characterising the construction of the PMG environment corresponds to the upper part of the core (0-115 cm depth, post-768±230 AD period; section B of Figure 2). This section is more dynamic than section A, and presents a mean sedimentation rate of 0.09 cm/yr with a dominance of marine sediments. A succession of important StE deposits contributed to the formation of a major marine sandy deposit behind the protecting dune, in place of the former mudflat. MGS and marine geochemical element concentrations increase, whereas continental elements decrease. These StE are dated at 768±230, 1325±80 and 1445±40 AD. The presence of *Salicorna* vegetation testifies to a tidal salt marsh environment. The 768±230 AD StE have deeply impacted the PMG lagoon, with a notable increase of MGS from 65-575 µm. The concentration of silicon (Si), Ca and Sr has been multiplied by 2, 6 and 3 while the concentration of Ti, zinc (Zn) and Fe has been divided

by 3, 4 and 5, respectively. Another StE hypothesis is debated with the marine deposit detected at 88 cm, dated at 1325±80 AD. This StE shows MGS (267-500 µm), Ca (17-45) and Sr (1-2.5) increases, with Fe (36-14) and Ti (4.3-1.4) decreases. The last StE hypothesis which could have led to the dune construction is dated at 1445±40 AD at 79 cm depth. This StE brought a 3 cm wide pebble into the PMG2017_3 core, interrupting the XRF geochemical analysis. MGS went from 50 to 500 µm. A lightness peak was detected, attesting the pebble remains. The CO_2 fall corresponds to the contact between the clay, rich in OM, and the overlying sands. Once the protecting dune was well formed, six more recent StE were also extracted at 1775±30, 1896, 1924, 1940, 1990 and 1999 AD, producing lower impacts in this recent and stabilised environment.

Throughout the TDC2017-3 core analysis extracted at Traicts du Croisic, three different stages are detected (Figure 3). The first stage is identified at the lowest part of the core (225-278 cm depth; section A) for the pre-930±50 AD period. The environment is calmer than in the overlying layer, which is mainly composed of clayey-silts, with a mean sedimentation rate of 0.08 cm/yr. This layer is interrupted by a marine deposit, formed from two past StE estimated at 522±60 and 661±55 AD. The MGS doubles between 260-250 cm during the first StE, and geochemical marine elements increase considerably. It is also the case of the CO_2 concentration, which rises due to shell remains. They were found during the second StE. Section A is interpreted as a low dynamic phase, attesting to the end of the Pen Bron sandy spit construction in a southern direction, which nowadays isolates the marsh from the sea. This spit was thinner than today which explains the clayey sediment between 245-225 cm. Geochemical analyses demonstrate that the mudflat clay has a terrestrial provenance, hence the presence of high Fe and Ti concentrations. In a second phase, between 930±50 AD (225 cm) and nearly 1920-1930 (20 cm), the protecting dune has strongly thickened (section B of Figure 3). An important marine input is detected during a high sedimentological dynamics

phase with a 0.22 cm/yr sedimentation rate. This second stage is identified through grain size and geochemical variations. OM values are low, whereas lightness increases with the bright colour of the marine sands, as opposed to continental clays. This high sedimentological accumulation, driven by seven StE identified at its base, strengthens the protecting dune. They have been dated at 973±44 (225 cm), 1083±30 (208 cm), 1054±30 (195 cm), 1270±30 (173 cm), 1279±30 (171 cm), 1300±35 (164 cm) and 1315±35 AD (161 cm). These seven StE all show notable increases in MGS, Sr/Ti, Sr and Ca and several exhibit decreases in Ti and MS. The oldest, detected at 225 cm depth, induced a CO_2 increase due to the *Verenupis decussata* shell detected in both the photography and radiography. The remaining part of this section, between 160-20 cm, is more homogeneous. MGS is more stable with low colorimetric, OM and SM variations. The dune became stabile during a calmer period. Geochemical analyses reveal Ca, Sr, Si and Sr/Ti increases for eight StE, with small impacts at 128, 122, 118, 107, 72, 64, 62 and 57 cm depths. The small concomitant MGS peaks confirm these hypotheses, which are dated, respectively, at 1429, 1460, 1470, 1504, 1628, 1665, 1678 and 1720±25-30 AD. In the third upper stage, a decrease of the sedimentological dynamics allowed this coastal system to return to a calmer environment (section C of Figure 3), dated post 1920-1930 AD. This 20 cm wide section is mainly composed of terrestrial clay with high CO_2, Zn, Fe and Ti concentrations. The environment returned to a mudflat as the dune became refined, with a sedimentation rate of 0.2 cm/yr. *Salicorna* vegetation observed in the lagoon testifies to a salt marsh environment, explaining the lower Ca or Sr values. A final marine deposit is detected in this section (at 10 cm), corresponding to a possible StE dated at 1974 AD. The photography and the Scopix highlight this StE, also characterised by notable variations of MGS (35-360 µm), lightness (45-50), Sr (0.8-1.6), Si (20-55), CO_2 (15-1%), Zn (0.2-0.07), Fe (43-10), Ti (4.5-1.7) and MS (1.2-0). The 1972 or 1977 storm events can be linked to this marine incursion.
